# A Novel Tongue Pressure Measurement Instrument with Wireless Mobile Application Control Function and Disposable Positioning Mouthpiece

**DOI:** 10.3390/diagnostics11030489

**Published:** 2021-03-10

**Authors:** Hsiu-Yueh Liu, Chun-Hung Chen, Chao-Hung Kuo, Ming-Chu Feng, Jen-Hao Chen, Hsuan-Wen Wang, Kun-Chun Chen, Chun-Li Lin

**Affiliations:** 1 Department of Oral Hygiene, College of Dental Medicine, Kaohsiung Medical University, Kaohsiung 80708, Taiwan; hyliu@kmu.edu.tw; 2 Department of Medical Research, Kaohsiung Medical University Hospital, Kaohsiung Medical University, Kaohsiung 80708, Taiwan; 3 Department of Neurology, Kaohsiung Municipal SiaoGang Hospital, Kaohsiung Medical University, Kaohsiung 81267, Taiwan; mp245@ms49.hinet.net; 4 Department of Neurology, Kaohsiung Medical University Hospital, Kaohsiung 80708, Taiwan; 5 Department of Internal Medicine, School of Medicine, College of Medicine, Kaohsiung Medical University, Kaohsiung 80708, Taiwan; kjh88kmu@gmail.com; 6 Division of Internal Medicine, Kaohsiung Municipal SiaoGang Hospital, Kaohsiung 81267, Taiwan; 7 Division of Internal Medicine, Kaohsiung Medical University Hospital, Kaohsiung 80708, Taiwan; 8 Department of Nursing, Kaohsiung Municipal SiaoGang Hospital, Kaohsiung Medical University, Kaohsiung 81267, Taiwan; mingchu0215@gmail.com; 9 School of Nursing, Fooyin University, Kaohsiung 83102, Taiwan; 10 School of Dentistry, College of Dental Medicine, Kaohsiung Medical University, Kaohsiung 80708, Taiwan; jehach@kmu.edu.tw; 11 Division of Prosthodontics, Department of Dentistry, Kaohsiung Medical University Hospital, Kaohsiung 80708, Taiwan; 12 Department of Biomedical Engineering, National Yang-Ming Chiao-Tung University, Hsinchu 30010, Taiwan; 411918@gmail.com (H.-W.W.); eq882812127002@gmail.com (K.-C.C.)

**Keywords:** tongue, tongue pressure, dysphagia, oral hypofunction, exercise, training

## Abstract

This study developed a novel chair-side tongue pressure (TP) measuring instrument with a disposable positioning mouthpiece controlled using a smartphone application (APP), denoted as the TP wireless application (TPWA). The mouthpiece was designed with a palate-shaped air balloon containing a tongue contact bump and a plastic bite positioning tube. Fatigue load testing was performed to evaluate mouthpiece durability by applying 700 displacement cycles (50 times a day for one week during training, with twice the safety factor) on the air balloon. The main component used in developing this instrument was a silicon pressure sensor equipped with wireless Bluetooth connection. Young (52 adults; mean age = 20.23 ± 2.17) and elderly (40 adults; mean age = 72.60 ± 7.03) individuals participated in the test with the new instrument, with the results compared to those of a commercial device. The TPWA mouthpiece fatigue test showed that mean response pressures were maintained at 12 kPa. No significant (*p* > 0.05) differences were found during testing repetitions 0–10 and 701–710. There were no significant differences in the maximum TP values presented between the test sequences using different instruments for young and elderly participants. The TPWA results showed that TP values gradually decreased with increasing age (40.77 kPa for young and 16.55 kPa for elderly participants). The maximum TP for males (43.51 kPa) was significantly larger than that for females (35.14 kPa) in the young group, but an opposite trend was seen in the elderly group (12.97 for males and 17.59 for females). Thus, this study developed a novel chair-side TP measurement instrument with Bluetooth wireless mobile application control. A durable positioning oral mouthpiece was approved for measuring pressure sufficiently, reliably, and precisely for TP screening.

## 1. Introduction

The tongue plays a critical role in food consumption. During the oral phase, food is controlled and mixed with saliva to form a bolus by the tongue. The tongue then moves the bolus into the pharynx and initiates the swallowing response. Tongue function is an important index for many age-related degenerative condition. Tongue strength is an important assessment reference indicator for swallowing function decline; dysphagia; sarcopenia; physical function; and pathophysiologic diagnosis, follow-up, and rehabilitation of nervous system and brain diseases that cause dysphagia [1,2,3,4,5,6,7,8,9]. Anterior tongue pressure (TP) measurement is commonly used to examine tongue function, owing to its quantifiable strength, endurance, and ability to be trained [4,10,11,12]. Maximum TP measurement is useful and important for evaluating tongue weakness for dysphagia in the oral stage or the oral preparation stage [6]. If the tongue weakens, liquids or solid food may enter the airway, triggering dysphagia complications such as choking, aspiration, pneumonia, malnutrition, and dehydration [2,13].

Many devices have been developed based on strain gauge manometry principles and are used to evaluate TP within the oral cavity, such as strain gauge manometry [14,15], force-sensitive resistors [16,17], and bulb pressure sensors [18,19]. The tongue bulb pressure sensor of the Iowa Oral Performance Instrument (IOPI Medical LLC, Carnation, WA, USA) is commonly used to measure tongue strength owing to its portability, pressure-sensing circuitry, peak hold function, and IOPI timer features [1]. A similar measuring device was developed by Yoshida et al., who modified the IOPI system into an auto-pressurization system called JMS (JMS TPM-01, JMS Co., Ltd., Japan) [9]. The probe was inflated with air to an initial pressure for calibration [9,20]. These commercial devices are used to diagnose tongue weakness, which has been associated with dysphagia [3,9]. These devices may also be used to increase tongue strength for dysphagia therapy [2,4,21,22].

Previous studies have reported that these devices have high costs for home use, requiring numerous connective components and air-filled bulbs that are prone to leaks, while the material properties can change with use and deformation [1,2,6]. A study used a single layer of gauze on a smooth air-filled bulb with a tongue bulb holder. Bulb slip was successfully reduced during measurement [23], however the tongue bulb holder is not currently being manufactured. Moreover, they recorded and calculated the data manually and lacked the ability for wireless connection or use with a mobile application (APP), because they were developed prior to smartphone development [1,5]. The exact location of the bulb is unstable and it slides easily into the user’s mouth when used with current market TP systems [1,5]. Different tongue and bulb contact positions and anterior teeth bite positions may affect the pressure measurement results. Based on previous studies, the contact position and stability between the tongue and the bulb in the mouth require long-term chair-side training to meet clinical measurement requirements. Hayashi et al. [20] designed a plastic pipe assembled from a rubber balloon and a tuberculin test syringe cylinder that can be held lightly between the user’s upper and lower central incisors to stabilize the pressurized parts in the oral cavity. If an efficient TP measurement system using a wireless connection cellular phone APP is connected to the host via network transmission which will help in rehabilitation training and follow-up of therapy effectiveness. Previous devices recorded or calculated data manually and lacked the ability to connect wirelessly or be used with a mobile application (APP) because they were developed prior to smartphone development [5]. Therefore, the main objective of this study is to develop a novel TP measure instrument controlled using APP visual operation and feedback through a Bluetooth connection to a smartphone based on bulb pressure sensors, including a sterilized and disposable positioning mouthpiece. This novel measurement device is called the tongue pressure wireless application (TPWA).It can be used to generate valid tongue pressure readings among young and older adults and is expected to be comparable to JMS devices.

## 2. Materials and Methods

### 2.1. Mouthpiece Device Design

**Figure 1:**
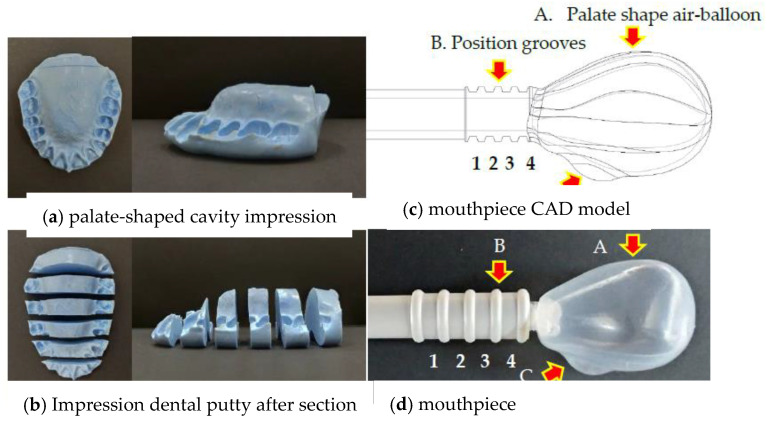

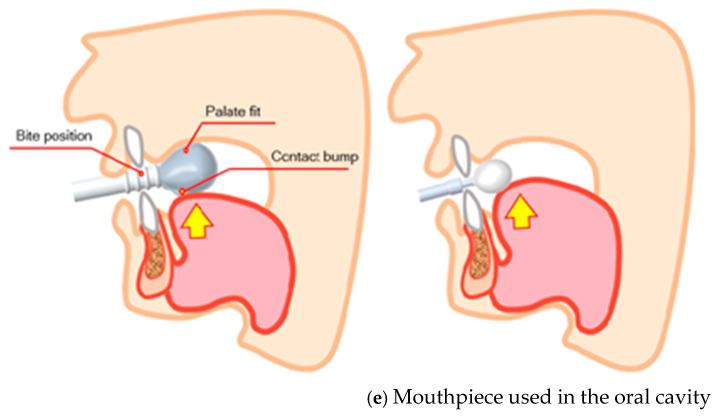
(**a**) Palate-shaped cavity impression with dental putty and (**b**) section contours with 5 mm cutting intervals of the impression putty. (**c**) Mouthpiece computer-aided design (CAD) model, (**d**) including a palate-shaped air balloon with a tongue contact bump and a plastic bite positioning tube. (**e**) Illustration of the new disposable positioning oral mouthpiece and an air-filled bulbs used in the oral cavity, shown in the left and right panels, respectively.

The mouthpiece features included a palate-shaped air balloon with a tongue contact bump and a plastic bite positioning tube. The palate anatomical shape was obtained through mouth cavity impressions using dental putty (Aquasil Soft Putty, Dentsply, Konstanz, Germany) material, filled between the palate and the tongue of each participant. Impression putty section contours with 5 mm cutting intervals were measured using the 2.5D image measurement system (ARCS Precision Technology Co., LTD, Taichung, Taiwan) (Figure 1a). All section contours of each impression model were imported into computer-aided design software (Creo 6.0, PTC Inc., Needham, MA, USA) to assemble the digital air balloon model, which resembled the palate anatomically. The finalized palate-shaped air balloon was then obtained by averaging the measurements from ten participants (five males and five females) with normal craniofacial morphology, without any history of orthodontic treatment, temporomandibular disorder, or orthodontic treatment, aged between 22 and 30 years old. A palate-shaped air balloon allowed the air balloon upper surface to stably fit with the anterior palate for TP testing. A bump (14 mm in diameter and 3 mm in depth) is an air-filled blister, which was referred to from the Kay device anterior sensor (Model 7120, KayPENTAX, Lincoln Park, NJ, USA) and was created in the anterior lower surface of the air balloon model (Figure 1b). The bump feature allowed the participant to have the same position for tongue contact in each test (Figure 1c). The plastic bite positioning tube was designed with four kinds of grooves with 1 mm spacing and 1.5 mm depth for upper and lower teeth bites, depending on the participant’s occlusal situation. The grooves allowed the participant to maintain the same biting position with mouthpiece placement in each test.

**Figure 2:**
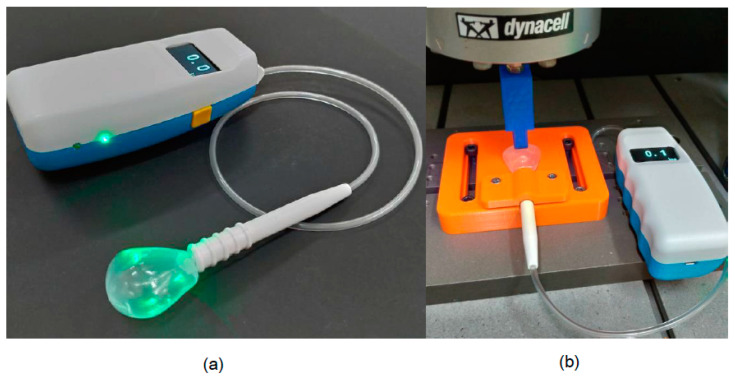
(**a**) The newly developed tongue pressure measurement instrument with wireless mobile application control function and disposable oral positioning mouthpiece. (**b**) Illustration of the dynamic load (fatigue) testing performed on the mouthpiece to evaluate its durability.

Low-density polyethylene (LDPE), a non-toxic material used for the air balloon, and acrylonitrile butadiene styrene (ABS), used for the plastic bite positioning tube, were fabricated using blow and injection molding, respectively, using an ISO13485 quality management system (Microware Precision Technology Co., Ltd., Taichung City, Taiwan). The mouthpiece was assembled using the LDPE palate-shaped air balloon, pipelines, and ABS plastic bite positioning tube (Figure 2a).

### 2.2. Mouthpiece Fatigue Testing

Dynamic load (fatigue) testing was performed on the mouthpiece to evaluate its durability. Three mouthpieces were clamped into a custom jig and fixed onto the Instron E3000 test machine with the axial load cell (Instron, Canton, MA, USA) (Figure 2b). The test frequency was 0.25 Hz, with a specific punch applied using a downward force on the air balloon bump to simulate TP. The punch bump was set to 10 mm and the number of cyclic displacements was 700, i.e., the air balloon received TP 50 times a day for one week during training, with twice the factor of safety. TP measurements were recorded every 100 cyclic displacement repetitions.

### 2.3. TP Instrument Development

**Figure 3:**
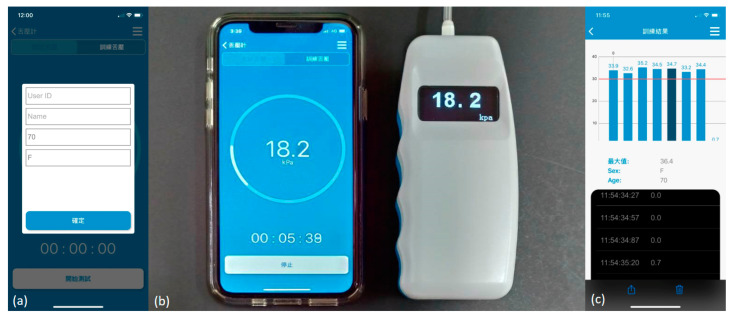
Screens of a novel tongue pressure measurement instrument: (**a**) measurement settings; (**b**) measurement screen in the smartphone and device; (**c**) results display screen.

The TPWA design principle was adopted using a silicon pressure sensor that can detect the air pressure range of 0–100 kPa, with a maximum 2.5% error. The thin-film resistors in the sensor were squeezed and the voltage variation was converted into a TP value when the air inside the air balloon was compressed by tongue contact (Figure 2a). The TP instrument was also equipped with a Bluetooth connection and a mobile device operating system. It can be connected to a smartphone wirelessly through Bluetooth and the parameters for TP measurement can be set directly through the APP. The recorded data can capture TP values every 0.5 s, which are displayed on the instrument monitor and the mobile phone screen simultaneously. The recorded data can be charted and stored in the device or uploaded to the cloud for real-time monitoring (Figure 3).

### 2.4. Participants and TP Measuring Method

Two healthy volunteer groups composed of 52 young (35 males and 17 females; age range = 18–27 years, mean age = 20.23 ± 2.17) and 40 older (9 males and 31 females; age range = 61–90 years, mean age = 72.60 ± 7.03) adults participated in the study approved, which was by the Ethics Committee (The Institutional Review Board; IRB) of Kaohsiung Medical University, Taiwan (protocol number: KMUHIRB-F(I)-20190104). Young participants had more than 28 natural teeth and they did not have any history of diseases that cause dysphagia, removable prosthodontic treatment, temporomandibular disorder, or orthodontic treatment. The recruitment criteria for older participants in this study were that they were living at home in the community, aged 60 years and over, and without any history of neurological diseases. We asked all participants to be examined by the same dentist to confirm that they had more than 20 teeth, and the Eichner index was A [5]. The exclusion criteria included those that had or were suspected of having dysphagia, as assessed by two tests (1) with a total Eating Assessment Tool (EAT-10) questionnaire score equal to or higher than 3 points [24]; and (2) participants who swallowed saliva less than three times within 30 s, as assessed by Repetitive Saliva Swallowing Test (RSST) test [5]. To determine whether there was an effect caused by the order of testing, both young and old participants were randomly assigned to different orders of the TP testing device.

The measurement procedures for maximum TP were similar using the new device and the JMS bulb. The two devices consist of a disposable oral probe, an infusion tube as a connector, and recording devices. The total measured time, number of measurement times, and duration in seconds for each measurement for the TPWA device can be set using the smartphone APP. The JMS system requires calibration to “zero” first at an initial pressure of 19.6 kPa to set the balloon diameter to approximately 18 mm with a volume of 3.7 mL by controlling an external recording box [6]. The TP is then measured through a probe connected to a pressure balloon for the new and the JMS devices. The participants sat in a relaxed position throughout testing. The mouthpiece of our new device and pressure balloon of the JMS were placed between the tongue and anterior section of the palate. Each participant needed to bite with free force on the first groove of the ABS plastic tube for positioning, whereby their tongue should have touched the air balloon bump, then they were asked to raise their tongue and press the balloon against the hard palate as firmly as possible when using the mouthpiece device. Maximum pressure values were obtained in real time and recorded from the TPWA and JMS devices by instructing participants to press the tongue to the palate as firmly as possible for 5 and 7 s, then the maximum value was recorded and the maximum TP was reported, respectively.

We conducted the triple TP tests for TPWA and JMS. Each participant rested for 30 s between each measurement. The mean value of the three measures was recorded as the maximum TP for each participant, as was done in the study by Utanohara et al. [5].

### 2.5. Statistical Analysis

JMP statistical software version 14 (SAS Institute, Cary, NC, USA) was used to perform inferential statistical analysis. A statistical paired *t*-test was performed to examine the differences in TP values before and after mouthpiece fatigue testing. Two sample *t*-tests were used to compare significant differences between different orders of TP testing and gender in both young and older populations. In order to analyze the relationship between age and tongue pressure, the participants in the older group were further categorized into 3 groups based on age (60–69 years, 70–79 years, and ≥80 years). ANOVA and post hoc Tukey’s HSD tests were performed. Statistical significance was set at 0.05.

## 3. Results

The results of our mouthpiece fatigue testing showed that the mean response pressures were maintained at 12 kPa during testing repetitions 0–10 and 701–710. The corresponding values between testing repetitions 0–10 and 701–710 times were found, with non-significant (all *p* > 0.05) differences for three individual samples (Table 1).

The corresponding values for young participants for test sequences (either TPWA–JMS and JMS–TPWA) were 42.31 ± 13.63 kPa/42.17 ± 12.23 kPa and 41.03 ± 10.21 kPa/39.64 ± 11.67 kPa, respectively. There was no significant difference in the data presented between the testing orders (Table 2). However, the maximum TP values for males were significantly larger than those for females, regardless of the testing order.

A similar trend was found for the older adults, i.e., no significant differences in the mean maximum TP values between the testing order were found and the corresponding values for TPWA–JMS and JMS–TPWA were 16.12 ± 8.78 kPa/19.12 ± 9.02 kPa and 20.00 ± 9.71 kPa/16.98 ± 9.75 kPa, respectively (Table 3). However, the maximum TP values for older males and females showed the opposite trend as those for the younger individuals—females had higher TP values than males, regardless of the testing order. Thus, with increasing age, the TP values gradually decreased for the older adults, which was also not related to testing order.

## 4. Discussion

The TPWA instrument we developed was updated to allow the user to directly operate it chair-side through a mobile phone with a wireless Bluetooth connection and APP program control based on the bulb pressure sensors. The bulb used by the current machines is difficult to position during the TP measurement process (Figure 1c) [1,2,6,20,25,26]. These factors may impact the accuracy of single measurements or the effectiveness of long-term rehabilitation and training. The flash diaphragm pressure inverter is not as user-friendly for routine diagnosis or treatment as bulb pressure sensor devices [6]. Our newly developed mouthpiece was designed with an anatomical palate-shaped air balloon rather than the previous bulb design to enhance stability in the mouth cavity. The bump at the anterior end of the lower air balloon surface rather than a smooth surface enables the participant to maintain a positive tongue contact position with the bite positioning grooves and allows the participant to maintain the same mouthpiece bite placement in each test.

The mouthpiece fatigue test results implied that patients can use our mouthpiece for long-term rehabilitation for a week, without damage and with stable quality. Our mouthpiece is designed to be disposable for a single screening. It also may provide for one-week therapy or rehabilitation training in the future. The period of use is about one week, and the number of times it can be used is about 350 times. The dynamic bump displacement test on the air balloon was performed 700 times, i.e., twice the safety factor consideration to ensure the stability of the air balloon material. The mouthpiece fatigue test showed that there were no significant differences in measured TP between 0–10 and 701–710 repetitions and the air balloon did not show any deformation or leaks in any samples. This guarantee is lacking in commercially available bulbs. As the new device is still in development, it is not possible to compute the exact device cost; however, a reusable mouthpiece would be relatively more economical than the current bulbs on the market and would be easily affordable for home or personal use.

In this study, young and older adults were randomly divided into subgroups with two different testing orders to eliminate or reduce the participant perception around which TP device was used first. The maximum TP results indicated that the values in young people were significantly higher than those in older adults, regardless of the order of machine use. This result is consistent with previously reported results [3]. We also found that the TP values of older adults were also reduced with age increasing when both devices were used, regardless of the order of machine use. Tongue strength decline with age could be due to age-related reduction in muscle mass [3,19,27]. The standard maximum TP values established by Utanohara et al. [5] for 60 and 70 years old were 38 kPa and 33 kPa, whereas the present data show nearly 50% reductions, regardless of the device used. However, the total study sample size in the study by Utanohara et al. was over four times (201) that of the current study (40). The large reductions in our study likely resulted from our insufficient sample size and large variability between older subjects. It is necessary to conduct a similar survey with a larger sample to establish more accurate TPs value for older subjects.

The testing order for the two pressure measurement instruments showed no differences between males and females. The maximum TP values for males were significantly higher than for females in the young participants. However, the opposite result was seen for the older adults, i.e., the females demonstrated significantly higher tongue strength than males. This could be because (1) the number of females was much higher than that of males (31:9) in the older group and (2) men experience larger losses in total muscle mass than women after 70 years of age [28]. Significant correlations were found between measurements made with the new device and the JMS device in young (Spearman’s *r* = 0.9084) and older adults (Spearman’s *r* = 0.7172). These results confirm the usability of the novel TPWA instrument. The grooves designed on the plastic bite positioning tube allowed the participants to maintain the same bite position in each test. This method is quite convenient and involves minimal patient burden [6]. However, this feature causes an inter-incisor separation of approximately 3–4 mm, which may affect the accuracy of the measurement. Nevertheless, the measured TP values were accurate; Solomon et al. indicated that there is no significant difference in tongue strength if jaw separation is 5 mm or less [20,29].

The tongue bump at the anterior lower surface of the air balloon mouthpiece allows participants to have the same tongue contact position in each test. We believe that the bump at the anterior lower surface of the air balloon should be more effective in long-term tongue training. However, the TPWA and JMS TP results showed no significant differences in the testing order or between young and older people, and may not fully present the true characteristics and importance. The reason might be that new air balloons were used in the TPWA and JMS instruments for single tests, without consideration of the slight differences in position function. The bump position can be adjusted lower in reference to the air balloon mouthpiece, similar to the KAY device anterior sensor, in order to understand the pressure differences between anterior, median, and posterior TP values more easily than with the KAY device [6]. Our results were similar to JMS, which confirmed that the new instrument is more convenient and effective for disease monitoring and long-term training related to dysphagia.

In recent decades, researchers have devoted themselves to studies of tongue pressure. Previous handheld bulb pressure measurement devices were easy for users to operate, but the testing position stability largely depended on the operator, resulting in a lack of comparability between different studies [30]. The JMS device has also been widely used for research studies on aging, frailty, sarcopenia, and dysphagia in Japan in the past 15 years and has been reported as having good validity [31,32]. From the results for these two devices, we did not find the above problem to exist in this study. According to the results in this study, which had a great correlation coefficient, the TP values obtained for the new device are almost identical to the values obtained by the JMS device for the anterior tongue. Therefore, the new device can be applied to generate as useful, meaningful, and valid tongue pressure values as JMS device in future studies.

## 5. Conclusions

A novel chair-side TP measurement instrument with Bluetooth wireless mobile application control was developed. The testing results proved this device to be able measure pressure sufficiently, reliably, and precisely for TP screening and observation of changes with age increases, almost equal to the JMS device. The attached disposable oral mouthpiece passed the fatigue life test and can be safely used for tongue strength training in long-term care in the future.

## Figures and Tables

**Table 1:** The results of our mouthpiece fatigue testing.

Testing Sample	Tongue Pressure during Testing Cycles (kPa)			*p*-Value	*p*-Value
	(Cycle 0–10)	(Cycle 300–310)	(Cycle 700–710)	(Cycle 0 v.s. 300)	(Cycle 0 v.s. 700)
	Mean(SD)	Mean(SD)	Mean(SD)		
Total	12.69(0.44)	12.40(0.11)	12.14(0.20)		
Sample 1	12.84(0.35)	12.31(1.04)	11.97(1.28)	0.14	0.05
Sample 2	13.04(0.76)	12.52(1.27)	12.36(1.35)	0.28	0.18
Sample 3	12.20(0.99)	12.36(1.34)	12.08(1.29)	0.76	0.82

**Table 2:** Tongue pressure values for young adults.

Testing Sample	Tongue Pressure during Testing Cycles (kPa)				*p*-Value		*p*-Value
	(Cycle 0–10)	(Cycle 300–310)		(Cycle 700–710)	(Cycle 0 v.s. 300)		(Cycle 0 v.s. 700)
	Mean (SD)	Mean (SD)		Mean (SD)			
Total	12.69(0.44)	12.40(0.11)		12.14(0.20)			
Sample 1	12.84(0.35)	12.31(1.04)		11.97(1.28)	0.14		0.05
Sample 2	13.04(0.76)	12.52(1.27)		12.36(1.35)	0.28		0.18
Sample 3	12.20(0.99)	12.36(1.34)		12.08(1.29)	0.76		0.82
**Variable**		**TPWA**		***p*-Value**	**JMS**		***p*-Value**
	***n***	**Mean**	**(SD)**		**Mean**	**(SD)**	
Total	52	40.77	(12.48)		41.51	(11.01)	
Test sequence							
TPWA–JMS	22	42.31	(13.63)	0.451	42.17	(12.23)	0.715
JMS–TPWA	30	39.64	(11.67)		41.03	(10.21)	
Gender							
Male	35	43.51	(11.72)	0.022	44.53	(10.59)	0.004
Female	17	35.14	(12.43)		35.30	(9.31)	

Abbreviations: TPWA–JMS: the testing sequences is TPWA first and then JMS; JMS–TPWA: the testing sequences is JMS first and then TPWA.

**Table 3:** Tongue pressure values for elders.

Variable		TPWA		*p*-Value	JMS		*p*-Value
	*n*	Mean	(SD)		Mean	(SD)	
Total	40	16.55	(9.17)		19.56	(9.26)	
Test sequence							
TPWA–JMS	20	16.12	(8.78)	0.772	19.12	(9.02)	0.768
JMS–TPWA	20	16.98	(9.75)		20.00	(9.71)	
Gender							
Male	9	12.97	(8.30)	0.187	14.91	(5.57)	0.087
Female	31	17.59	(9.27)		20.91	(9.74)	
Age							
60–69 yrs	18	19.66	(10.61)	0.086	22.89	(10.13)	0.118
70–79 yrs	15	15.40	(6.87)		17.02	6.61	
≥80 yrs	7	11.01	(7.01)		16.43	(10.20)	

Abbreviations: TPWA–JMS: the testing sequences is TPWA first and then JMS; JMS–TPWA: the testing sequences is JMS first and then TPWA.
